# Clinicopathological Characteristics of Extrapulmonary Neuroendocrine Carcinomas: Treatment Responses and Survival Outcomes: Single-Center Experience

**DOI:** 10.3390/jcm14072264

**Published:** 2025-03-26

**Authors:** Harun Muğlu, Erdem Sünger, Maral Martin Mıldanoğlu, Ebru Engin Delipoyraz, Mehmet Haluk Yücel, Hakan Özçelik, Jamshid Hamdard, Özgür Açıkgöz, Ömer Fatih Ölmez, Özcan Yıldız, Ahmet Bilici

**Affiliations:** Department of Medical Oncology, Faculty of Medicine, Medipol University, Istanbul 34214, Türkiye; erdemsunger@gmail.com (E.S.); mmildanoglu@gmail.com (M.M.M.); drebruengin@gmail.com (E.E.D.); mhalukyucel@gmail.com (M.H.Y.); hknozcelikk@gmail.com (H.Ö.); jamshidhamdard@hotmail.com (J.H.); ozgur_acikgoz@yahoo.com (Ö.A.); omerfatih.olmez@medipol.com.tr (Ö.F.Ö.); ozcanyildiz71@gmail.com (Ö.Y.); ahmetknower@yahoo.com (A.B.)

**Keywords:** extrapulmonary neuroendocrine carcinoma, platinum-based chemotherapy, survival analysis, Ki-67 index, surgical intervention

## Abstract

** Background/Objectives**: Extrapulmonary neuroendocrine carcinomas (EP-NECs) are rare, aggressive malignancies with no standardized treatment approach. Although platinum-based chemotherapy is considered the first-line therapy, overall survival (OS) and progression-free survival (PFS) remain limited. This study aims to evaluate the clinical and pathological characteristics of EP-NEC patients, their treatment responses, and survival outcomes. **Methods**: This retrospective observational study included 29 EP-NEC patients diagnosed and followed between 2015 and 2024. Clinical and demographic data, tumor localization, disease stage, administered treatments, and survival outcomes were analyzed. Kaplan–Meier survival analysis was used to assess OS and PFS, with subgroup comparisons performed via the log-rank test. **Results**: The most common primary tumor sites were the pancreas (21%), prostate (17%), and cervix (14%). At diagnosis, 55.2% of patients had metastatic disease. First-line platinum-based chemotherapy achieved an objective response rate of 82.1%, with a median PFS of 8.16 months and a median OS of 14.16 months. Surgical intervention significantly improved survival (*p* = 0.020), while a high Ki-67 proliferation index (>80%) was associated with worse PFS (*p* = 0.032). Other factors, including smoking status and liver-directed therapies, had no significant impact on survival. **Conclusions**: EP-NECs present with a poor prognosis despite platinum-based chemotherapy achieving high response rates. Surgical resection improves survival outcomes, whereas high Ki-67 expression is associated with a worse prognosis. These findings highlight the need for further research into novel therapeutic strategies for EP-NECs.

## 1. Introduction

Extrapulmonary neuroendocrine carcinomas (EP-NECs) are extremely rare and can develop in various parts of the gastrointestinal (GI) tract. In the squamous-lined regions of the GI tract, such as the esophagus and anus, they predominantly exhibit small-cell histology [1]. Within the jejunum and ileum, EP-NECs account for only 1% of all neuroendocrine tumors [2]. Additionally, extrapulmonary small-cell carcinomas can arise in the bladder (0.3–1% of cases), cervix (1% of cases), and prostate (2% of cases) [3]. These tumors are characterized by rapid proliferation, high mitotic rates, and poor differentiation, leading to limited treatment options and poor prognosis [4,5].

Extrapulmonary neuroendocrine carcinomas (EP-NECs) are aggressive malignancies characterized by the expression of neuroendocrine (NE) markers, including chromogranin A, synaptophysin, and the neuron cell adhesion molecule, as well as a high proliferative index (Ki-67 > 55%). Unlike well-differentiated neuroendocrine tumors (WD-NETs), which display an organoid-like growth pattern, EP-NECs exhibit poor differentiation. Histologically, they resemble pulmonary NECs and can present with either “small-cell” morphology—characterized by compact sheets of cells with scant cytoplasm, fusiform nuclei, minimal nucleoli, and finely granular chromatin—or “large-cell” morphology, which consists of trabecular or nest-like arrangements of round to polygonal cells, moderate cytoplasm, and large nuclei with prominent nucleoli and vesicular chromatin [6,7,8,9].

Neuroendocrine neoplasms (NENs) encompass a spectrum of tumors ranging from WD-NETs to poorly differentiated neuroendocrine carcinomas (NECs), which include both pulmonary and extrapulmonary subtypes. High-grade neuroendocrine carcinomas (HG-NECs) are aggressive malignancies, characterized by a high proliferative index (Ki-67 > 55%), poor differentiation, and rapid progression, distinguishing them from well-differentiated NETs, which have lower mitotic rates, better differentiation, and relatively favorable prognoses [10].

Unlike HG-NECs, well-differentiated NETs are typically slow-growing neoplasms with a more organized histopathological structure, lower Ki-67 proliferation index, and an ability to retain hormone-secreting functionality in some cases. These tumors are commonly found in the gastrointestinal tract and pancreas, where they may exhibit somatostatin receptor expression, allowing for targeted therapies such as somatostatin analogs and peptide receptor radionuclide therapy [5,11].

Additionally, EP-NECs exhibit distinct biological and clinical differences compared to their pulmonary counterparts. While small-cell lung carcinoma (SCLC) and large-cell neuroendocrine carcinoma (LCNEC) frequently harbor TP53 and RB1 mutations, EP-NECs demonstrate greater genetic heterogeneity, often involving KRAS, PIK3CA, or MEN1 alterations, which may influence treatment response [12,13,14]. This heterogeneity is reflected in the clinical behavior of EP-NECs, as their response to platinum-based chemotherapy is often less favorable than that of pulmonary NECs. Unlike pulmonary NECs, which frequently exhibit a near-uniform sensitivity to platinum-etoposide chemotherapy, EP-NECs show variable responses depending on their anatomical origin and molecular profile. While SCLC and LCNEC typically demonstrate initial high response rates to first-line chemotherapy, EP-NECs tend to have lower response rates and shorter progression-free survival, emphasizing their biological diversity [15,16].

Despite these differences, treatment paradigms for EP-NECs are largely extrapolated from pulmonary NECs, with platinum-based chemotherapy being the standard of care. However, emerging evidence suggests that response rates and survival outcomes differ, emphasizing the need for tailored therapeutic approaches for EP-NECs [17].

Recent genomic analyses indicate that EP-NECs may benefit from alternative therapeutic strategies, including molecularly targeted agents and immunotherapy, particularly in subgroups exhibiting specific alterations such as BRAF or MSI-high status [18].

Although platinum-based regimens remain the first-line standard, there is a growing interest in personalized treatment approaches that integrate molecular profiling to optimize therapeutic efficacy [19].

At the time of diagnosis, most patients with EP-NECs already have metastatic disease, with a median survival of less than a year [20]. The treatment landscape for these patients is highly limited; while surgery remains the preferred option in localized cases, platinum-based chemotherapy has been the standard first-line palliative approach for over three decades [1]. Although up to ~70% of patients demonstrate initial radiological responses to platinum-based chemotherapy, disease progression typically occurs within 4 to 9 months, leading to a short median progression-free survival (PFS) [21,22]. Alternative chemotherapy regimens have been explored in retrospective analyses and non-randomized studies following platinum-based treatment failure, but no universally accepted second-line standard has been established to date [23].

Despite their histological similarities to SCLC, EP-NECs lack a standardized treatment approach. Current therapeutic strategies primarily rely on platinum-based chemotherapy, mirroring SCLC treatment guidelines [7,24,25]. However, retrospective studies indicate that response rates in EP-NECs are lower compared to SCLC, with a median progression-free survival (PFS) of 4–9 months and an overall survival (OS) ranging from 5 to 16 months [16,25,26]. Recent advancements in molecular profiling have highlighted the genetic heterogeneity of EP-NECs, distinguishing them from their pulmonary counterparts. Key genetic alterations, including TP53, RB1, MYCN amplification, and BRAF mutations, suggest potential therapeutic targets beyond conventional chemotherapy [18]. Although platinum-based chemotherapy remains the cornerstone of first-line treatment for EP-NECs, its durability in disease control is limited, highlighting the need for further research into molecularly targeted agents and immunotherapeutic strategies [13,27].

Historically, EP-NECs have been managed as a single disease entity; however, analyses of large datasets have revealed considerable heterogeneity in both survival outcomes and treatment responses, suggesting underlying biological diversity within this group of tumors [20,28]. Among the key prognostic indicators, the anatomical origin of the tumor and the Ki-67 proliferation index—specifically a threshold of 55%—have been identified as critical factors. Patients with a Ki-67 index below 55% tend to have significantly longer overall survival (OS), yet they also demonstrate a reduced likelihood of responding to platinum-based chemotherapy when compared to those with a Ki-67 index of 55% or higher [28,29].

This study aims to evaluate the clinical characteristics of EP-NEC patients, assess the treatment responses, and the identify prognostic factors associated with survival outcomes. By integrating the findings from contemporary literature, this research seeks to highlight the urgent need for novel therapeutic strategies beyond traditional chemotherapy.

## 2. Materials and Methods

### 2.1. Study Design

This study was designed as a retrospective observational analysis.

### 2.2. Study Setting and Sample

A total of 29 patients diagnosed with EP-NEC between 2015 and 2024 and followed up during this period were included in the study.

### 2.3. Inclusion and Exclusion Criteria

Patients with pulmonary neuroendocrine carcinoma or those with histopathological evidence of mixed tumors were excluded. This study included only patients with pure neuroendocrine carcinoma (NEC). Patients with adenocarcinoma (AC) or mixed histology (e.g., NEC + AC components) were excluded from the study. All histopathological diagnoses were confirmed through a centralized pathological review to ensure diagnostic accuracy and consistency.

### 2.4. Data Collection

Clinical and demographic data, date of diagnosis, disease stage at presentation, administered treatments, progression timelines, and survival outcomes were reviewed. Demographic data, including age, sex, smoking history, and Eastern Cooperative Oncology Group (ECOG) performance status (PS), were recorded. Primary tumor localization, disease stage at diagnosis (local, locoregional, metastatic), and metastatic sites (liver, bone, lymph nodes, etc.) were evaluated. The administration of surgery, chemotherapy, radiotherapy, and immunotherapy, along with treatment responses, were documented.

### 2.5. Statistical Analysis

Statistical analyses were conducted using SPSS v27.0 (IBM Corp., Armonk, NY, USA). For categorical data, the results were summarized in terms of the count distributions and relative proportions. Survival-related continuous variables were expressed using both mean and median values. To assess the impact of clinicopathological characteristics on survival outcomes, univariate and multivariate Cox proportional hazards models were applied, with the type I error rate fixed at 5%. Furthermore, overall survival (OS) and progression-free survival (PFS) were analyzed through Kaplan–Meier survival curves, with comparisons made using the log-rank test.

### 2.6. Ethical Considerations

This study was reviewed and approved by the Non-Interventional Clinical Research Ethics Committee of Istanbul Medipol University (Approval No: 150, Date: 6 February 2025). All research procedures were conducted in accordance with the ethical principles outlined in the Declaration of Helsinki.

## 3. Results

This study included 29 patients diagnosed with EP-NECs, with a nearly equal gender distribution and a median age of 60.14 years (range: 29–82). At initial diagnosis, 55.2% of patients had metastatic disease, while 10.3% had localized and 34.5% had locoregional disease. The majority of patients (69%) were non-smokers. The Ki-67 proliferation index was greater than 80% in 75% of patients. The treatment approaches varied, including curative-intent radiotherapy (39.3%), concurrent chemoradiotherapy (25%), liver-directed therapies (6.9%), and surgical resection (31%). All patients received platinum-based chemotherapy, with 51.7% receiving cisplatin–etoposide and 48.3% receiving carboplatin–etoposide. Detailed demographic and clinical characteristics are summarized in Table 1.

**Figure 1:**
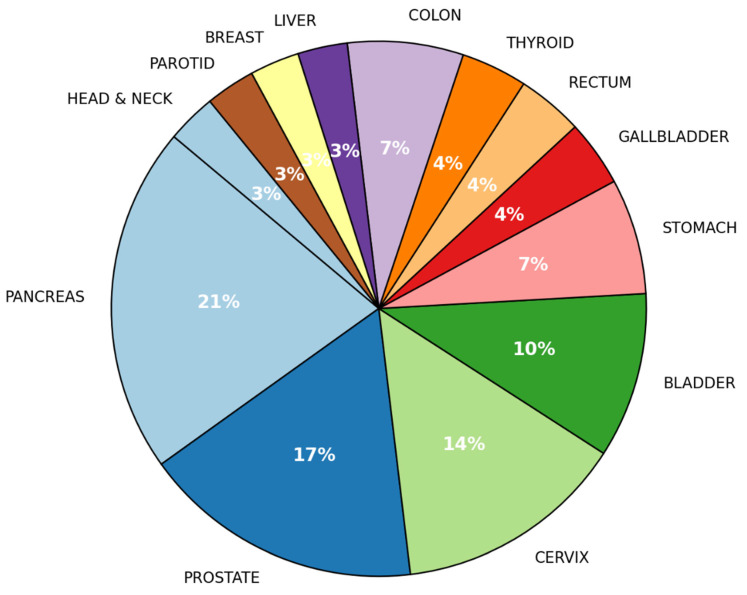
Location of the primary tumor in all cases (local/regional and metastatic disease).

**Figure 2:**
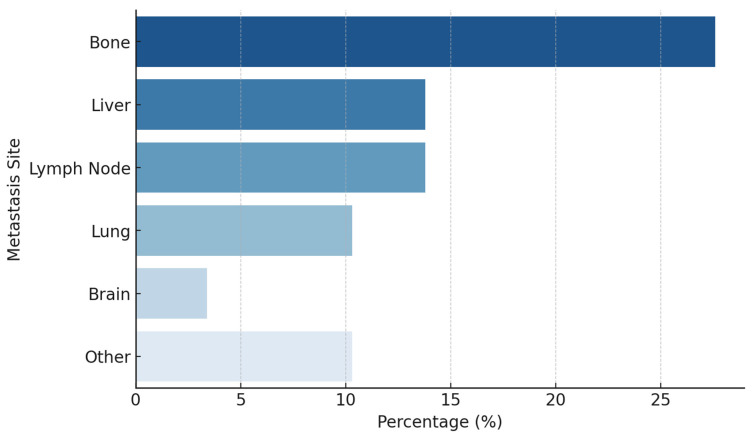
Frequency of metastasis sites.

The most common primary tumor site was the pancreas (21%), followed by the prostate (17%) and cervix (14%). Other sites included the bladder (10%), stomach (7%), colon (4%), and liver (4%). Less common primary tumor locations involved the rectum, gallbladder, thyroid, breast, parotid gland, and head-neck region, each accounting for approximately 3–4% of cases (Figure 1). Among the 16 individuals identified with metastatic disease, the predominant metastatic sites included the skeletal system (27.6%), hepatic tissue (13.8%), lymphatic structures (13.8%), pulmonary regions (10.3%), and the central nervous system (3.4%) (Figure 2).

**Figure 3:**
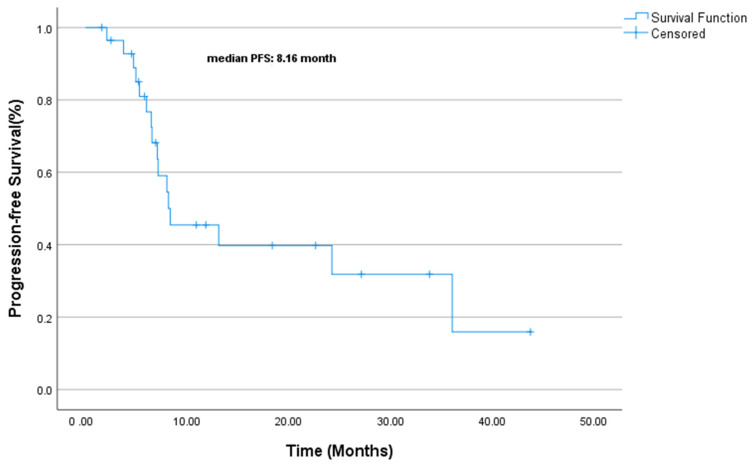
Progression-free survival.

**Figure 4:**
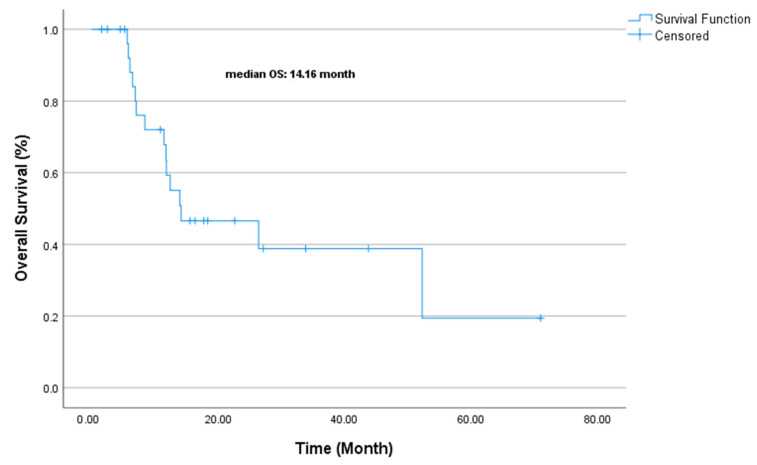
Overall survival.

At the median follow-up of 25.6 months (range: 6.5–33.2), the median PFS was 8.16 months (CI 95% 1.88–14.45, Figure 3). The median OS was 14.16 months (CI 95% 5.92–31.31, Figure 4). The univariate analysis of PFS and OS identified key prognostic factors. For PFS, ECOG performance status (*p* = 0.005), surgical history (*p* = 0.020), and Ki-67 index (*p* = 0.032) were statistically significant prognostic factors. In other words, poor ECOG PS (2 vs. 0–1) and lack of surgery were associated with a worse PFS, while patients with a lower Ki-67 (<80%) had slightly better outcomes. Although not statistically significant, a first-line chemotherapy choice (cisplatin–etoposide vs. carboplatin–etoposide) showed a numerical trend favoring cisplatin–etoposide (13.1 vs. 7.1 months, *p* = 0.182).

The response to first-line therapy was notable, with a complete response (CR) in 12 patients (42.9%), partial response (PR) in 11 patients (39.3%), and progressive disease (PD) in 5 patients (17.9%), yielding an overall objective response rate (ORR) of 82.1%. Second-line chemotherapy was administered to 15 patients (51.7%) and included irinotecan (17.2%), cisplatin–etoposide (6.9%), and other regimens, such as paclitaxel and oxaliplatin plus capecitabine (CAPOX) (17.2%). The response rates for second-line therapy showed PR in three patients (10.3%), stable disease (SD) in one patient (3.4%), and PD in seven patients (24.1%). Third-line chemotherapy was given to 10 patients (34.5%) with a median of three cycles per patient. Immunotherapy was administered to one patients (10.3%), where one patient exhibited stable disease (SD) and another experienced disease progression (Table 2).

Furthermore, 1 L therapy demonstrated a significantly higher ORR compared to 2 L therapy. The ORR for 1 L therapy was 82.1%, while for 2 L therapy, it was notably lower at 10.3%. For patients receiving 1 L chemotherapy, the median PFS was 8.17 months (95% CI: 1.88–14.45), and the median OS was 14.17 months (95% CI: 0.00–31.31). Within this group, 42.9% of patients achieved a CR, 39.3% had a PR, and 17.9% experienced disease progression (Table 2).

**Figure 5:**
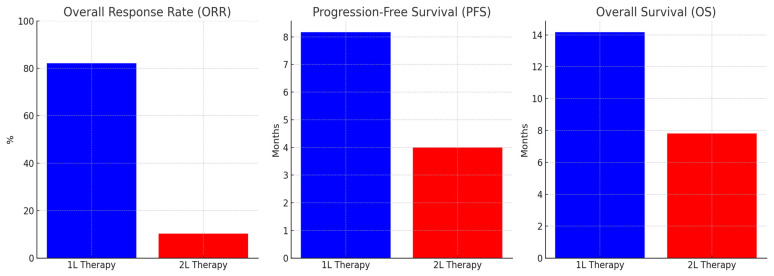
Comparison of 1 L vs. 2 L chemotherapy: ORR, PFS, and OS.

In contrast, 2 L chemotherapy resulted in a median PFS of 4 months and a median OS of 7.8 months. PR was observed in only 10.3% of patients, SD in 3.4%, and PD in 24.1%. Among 2 L regimens, irinotecan-based treatments exhibited a slightly better PFS (4.8 months) compared to taxane-based regimens (3.2 months), though this difference did not reach statistical significance (*p* = 0.065) (Figure 5).

For OS, no variables reached statistical significance, but first-line chemotherapy (cisplatin–etoposide vs. carboplatin–etoposide) and disease stage showed numerically meaningful differences. Patients receiving cisplatin–etoposide had a longer OS (26.4 vs. 11.7 months, *p* = 0.180), and those with local disease survived longer (52.2 months) than those with locoregional (12.4 months) or metastatic disease (11.8 months) (*p* = 0.520).

To further investigate the prognostic factors, the survival outcomes were stratified based on tumor localization, Ki-67 proliferation index, and treatment modality.

When stratified by anatomical origin, no statistically significant difference was observed between gastrointestinal (GI) and genitourinary (GU) primary sites in terms of the OS and PFS (*p* = 0.278). However, a notable trend emerged regarding PFS. Patients with pancreatic EP-NEC had a shorter median PFS of 6.2 months, whereas those with GU-origin tumors had a median PFS of 9.8 months. Among patients with GU-origin tumors, those who underwent surgical resection had a significantly longer median PFS (12.4 months) compared to non-surgical cases (7.5 months). This suggests that tumor localization and treatment approaches may influence disease progression.

**Figure 6:**
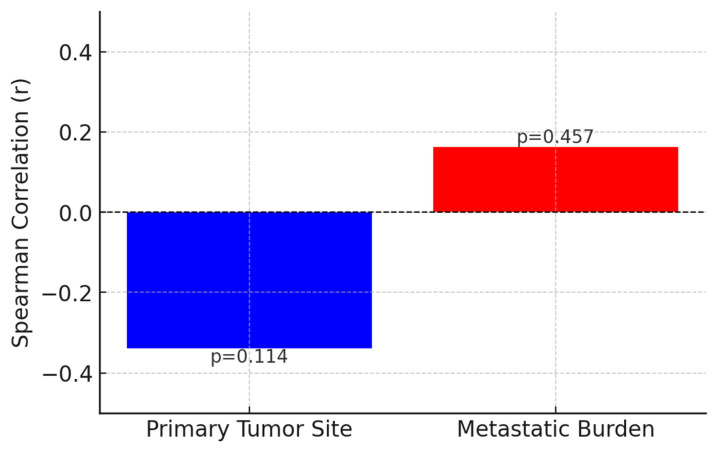
Correlation of Ki-67 index with tumor site and metastatic burden.

Additionally, to evaluate the potential correlation between tumor grade and both the primary tumor site and metastatic burden, a Spearman correlation analysis was conducted. The results indicated a weak negative correlation between the Ki-67 proliferation index and the primary tumor site (r = −0.339, *p* = 0.114), which did not reach statistical significance. Similarly, a weak positive correlation was observed between the Ki-67 index and metastatic burden, assessed by the number of metastatic sites (r = 0.163, *p* = 0.457), but this relationship was also not statistically significant. These findings suggest that the proliferative activity of EP-NECs, as measured by Ki-67 expression, does not appear to be strongly influenced by the tumor’s anatomical origin or the extent of metastatic disease at diagnosis. While the tumor site and metastatic burden are critical prognostic factors in many malignancies, the lack of significant correlation in this study implies that tumor proliferation may be governed by intrinsic biological mechanisms rather than by localization or metastatic extent (Figure 6).

A Kaplan–Meier analysis was performed to evaluate the impact of gender on PFS. The median PFS was calculated as 8.3 months for male patients and 8.0 months for female patients. The log-rank test results indicated no statistically significant difference in PFS between genders (*p* = 0.747). Similarly, no statistically significant difference was observed in OS, with median OS durations of 11.8 months in male patients and 26.4 months in female patients (*p* = 0.451). Notably, gender-specific primary tumor distributions were evident, with prostate NECs exclusively observed in males (17.2%) and gynecologic NECs, particularly cervical involvement, occurring solely in females (13.8%). In contrast, other primary sites, including pancreatic (21%), gastric (7%), colorectal (4%), and biliary NECs (4%), were distributed across both genders without a significant predilection. These findings suggest that while primary tumor localization varies by gender, gender itself is not an independent prognostic factor for survival outcomes in EP-NEC patients.

Multivariate analysis revealed that the Ki-67 index, surgical intervention, and immunotherapy status were independent prognostic factors for PFS. Patients with a high Ki-67 index (≥80%) had significantly worse PFS (*p* = 0.000), indicating its role in tumor aggressiveness. Surgical resection was associated with a significant improvement in PFS (*p* = 0.030), emphasizing the potential survival benefit of surgery in selected patients. Additionally, patients who did not receive ICIs had an increased risk of progression (*p* = 0.035).

In contrast, ECOG PS (*p* = 0.463) and the choice of a first-line chemotherapy regimen (cisplatin–etoposide vs. carboplatin–etoposide, *p* = 0.475) did not show significant associations with PFS. These findings underscore the impact of tumor biology and treatment approach on disease progression (Table 3).

Multivariate analysis revealed that none of the evaluated factors were independent predictors of OS (*p* > 0.05). However, certain variables exhibited numerical trends. The choice of first-line chemotherapy (cisplatin–etoposide vs. carboplatin–etoposide) showed a tendency towards improved OS in patients receiving cisplatin–etoposide (26.4 vs. 11.7 months, *p* = 0.331), though this was not statistically significant. Similarly, patients undergoing surgical intervention demonstrated better survival outcomes (26.4 vs. 11.8 months, *p* = 0.705), yet the effect was not statistically significant in the multivariate model. The Ki-67 index was not a significant predictor of OS (*p* = 0.645). These findings suggest that while surgical resection and chemotherapy choice may influence survival, their effects were not robust enough to reach statistical significance in the current dataset (Table 4).

Patients with localized disease demonstrated a median OS of 24.2 months, whereas those with metastatic disease had significantly shorter survival at 11.8 months (*p* = 0.520). A similar trend was observed for PFS, with localized disease exhibiting a median PFS of 24.2 months compared to 8.0 months in metastatic cases. No statistically significant difference in OS and PFS was identified between GI and GU tumor origins (*p* = 0.278), although a trend toward shorter PFS was noted in pancreatic EP-NECs (Table 3).

Tumor grade, as assessed by the Ki-67 proliferation index, was a significant prognostic factor. Patients with a Ki-67 index < 80% had a notably longer median PFS (36.0 months) compared to those with a Ki-67 ≥ 80% (8.0 months, *p* = 0.032), reinforcing the aggressive nature of highly proliferative tumors.

The treatment modality also influenced survival outcomes. Patients who underwent surgical resection had significantly improved OS (26.4 vs. 11.8 months, *p* = 0.020) and PFS. First-line platinum-based chemotherapy regimens (cisplatin–etoposide vs. carboplatin–etoposide) did not show a statistically significant difference in OS (*p* = 0.180); however, a numerical advantage was noted for cisplatin–etoposide (26.4 vs. 11.7 months) (Table 4).

These findings suggest that ECOG-PS, surgical intervention, and the tumor proliferation index significantly influenced PFS, while chemotherapy choice and disease stage may have an impact on OS. These findings highlight the prognostic impact of tumor localization, as well as the proliferation rate, and treatment approach in EP-NEC patients, emphasizing the need for individualized therapeutic strategies.

Detailed results of the univariate analysis for PFS and OS are available in Table 3 and Table 4, respectively.

## 4. Discussion

EP-NECs are rare and aggressive malignancies with limited treatment options. Despite the use of platinum-based chemotherapy as the first-line treatment, survival outcomes remain poor. Our study, consistent with the existing literature, highlights the challenges in managing EP-NECs and underscores the need for more effective therapeutic strategies.

EP-NECs and SCLC share histopathological similarities, including a high Ki-67 index (>55%) and neuroendocrine marker expression. However, their clinical behavior, treatment responses, and OS differ significantly [19,30].

EP-NECs exhibit greater biological heterogeneity than SCLC. While SCLC is commonly driven by TP53 and RB1 mutations, EP-NECs display a broader genetic spectrum, including KRAS, PIK3CA, and MEN1 mutations [31,32].

This may contribute to their lower response rates to platinum-etoposide chemotherapy. Survival outcomes also differ. Large cohort studies report a median OS of 8–14 months for metastatic EP-NECs, while SCLC patients achieve 10–18 months under similar treatment. EP-NECs tend to progress more rapidly, with a median PFS of 8.16 months, compared to 9–12 months in SCLC [33].

Current management largely follows SCLC protocols due to shared histopathological features, yet accumulating evidence highlights the need for a more tailored strategy. The Ki-67 proliferation index plays a crucial role in guiding treatment decisions and prognosis [3].

For patients with a low Ki-67 index (<20%), an indolent disease course is expected, and treatment is often limited to observation, somatostatin analogs (SSAs), tyrosine kinase inhibitors, locoregional treatment, or surgical resection if feasible. In cases of progression, mTOR inhibitors such as everolimus can be considered. Chemotherapy is generally avoided unless there is evidence of rapid disease progression [5].

Patients with an intermediate Ki-67 index (20–55%) require a more aggressive approach, with platinum-based chemotherapy (cisplatin or carboplatin + etoposide) as the first-line treatment. Irinotecan-based regimens (FOLFIRI) and paclitaxel-based chemotherapy have demonstrated efficacy in the second-line setting, particularly in cases with histological variations such as squamous differentiation. Localized tumors that are unresectable may benefit from radiation therapy for disease control. Molecular profiling should be performed in progressive cases to explore targeted treatment options [4,6].

In high Ki-67 index tumors (>55%), a highly aggressive disease course necessitates systemic chemotherapy as the cornerstone of treatment. Platinum-etoposide remains the standard first-line regimen, with irinotecan or paclitaxel-based therapies recommended in the second-line setting [7]. Immunotherapy, particularly checkpoint inhibitors, such as pembrolizumab or nivolumab, should be considered for patients with high PD-L1 expression, microsatellite instability (MSI-high), or high tumor mutational burden (TMB). Novel strategies, including DLL3-targeting antibody-drug conjugates, are currently under investigation for treatment-refractory cases [31,34,35,36].

Surgical intervention is beneficial in select cases of localized disease, often improving survival when combined with adjuvant chemotherapy or radiation. However, in metastatic disease, systemic treatment remains the primary therapeutic approach. Molecular profiling should be incorporated into clinical practice to identify potential therapeutic targets in refractory cases.

Surgical resection offers survival benefits in EP-NEC, unlike in SCLC, where it plays a minimal role [35]. Additionally, molecularly targeted therapies and immunotherapy may be beneficial for EP-NECs with MSI-high or TMB-high. Overall, while EP-NECs and SCLC share pathological features, their prognostic differences necessitate EP-NEC-specific treatment approaches rather than simply adapting SCLC treatment strategies [19].

Treatment regimens were selected based on internationally recognized guidelines and previously published studies on the management of EP-NEC. The European Neuroendocrine Tumor Society (ENETS) and the North American Neuroendocrine Tumor Society (NANETS) recommend platinum-based chemotherapy, specifically cisplatin or carboplatin in combination with etoposide, as the standard first-line treatment for patients with poorly differentiated neuroendocrine carcinomas due to their aggressive nature and high proliferation index [4,37].

Several retrospective and prospective studies have demonstrated that patients with a high Ki-67 proliferation index (≥55%) and extensive disease respond better to platinum-based chemotherapy, achieving an ORR of 40–70% and OS of approximately 11–16 months [38]. Platinum-based chemotherapy is believed to be more effective in these cases due to the rapid tumor proliferation and greater dependency on DNA repair pathways, which are disrupted by platinum agents, including temozolomide alone or in combination with capecitabine (CAPTEM), which are generally considered for second-line treatment, particularly in patients with a lower Ki-67 index or contraindications to platinum agents. The rationale for not first-line treatment is based on its lower objective response rates (15–30%) compared to platinum-based chemotherapy and its slower onset of action, which may not be suitable for patients with aggressive, rapidly progressing tumors. Furthermore, temozolomide’s efficacy has been shmethylguanine-DNA methyltransferase (MGMT) expression, where tumors with low MGMT levels exhibit better responses, making it a more selective treatment choice [39].

Based on these considerations, our study prioritized platinum–etoposide-based treatment, while temozolomide-based regimens were reserved for patients who exhibited platinum resistance or intolerance. This approach aligns with the current clinical practice recommendations and ensures that patients receive the most effective treatment tailored to their disease biology and clinical presentation.

In our cohort, first-line platinum-based chemotherapy demonstrated a high ORR (82.1%), compatible with prior reports that show initial response rates of up to 70–80% [19]. However, disease progression occurred rapidly in our study, with a median PFS of 8.16 months and median OS of 14.16 months, which are relatively favorable compared to other studies that reported PFS ranging from 5.83 to 9 months and OS between 13.6 and 16 months [19,40]. This variability may stem from differences in patient characteristics, treatment approaches, and disease burden at the diagnosis. Notably, patients who underwent surgical resection had significantly improved survival outcomes (*p* = 0.02), supporting the notion that resection should be considered in selected cases where feasible [41].

Our study also evaluated treatment beyond first-line therapy. Second-line chemotherapy resulted in a partial response in only 10.3% of patients, while 24.1% had disease progression, suggesting diminishing efficacy with successive lines of treatment. Third-line therapy yielded no partial responses, with only 6.9% of patients achieving stable disease, indicating a critical need for alternative approaches in refractory EP-NEC cases.

Our findings indicate that 1 L chemotherapy remains the most effective systemic treatment for EP-NEC, achieving superior response rates and longer survival compared to 2 L therapy. The significant decline in response rates and survival outcomes in 2 L treatment suggests that disease progression is associated with increasing chemoresistance [42]. This aligns with previous studies indicating that patients with high-grade neuroendocrine carcinomas experience diminishing benefits with successive lines of therapy. The low ORR observed with 2 L therapy highlights the urgent need for alternative treatment strategies in refractory EP-NEC [39]. Current therapeutic options beyond 1 L therapy, including irinotecan- and taxane-based regimens, demonstrate limited efficacy. The lack of significant survival differences between these regimens underscores the necessity of exploring novel approaches, such as molecularly targeted therapies or immunotherapy, in relapsed cases.

Our findings indicate that tumor localization may influence survival outcomes, with pancreatic EP-NECs exhibiting a more aggressive disease course, reflected by a shorter median PFS. The relatively longer PFS observed in GU-origin tumors, particularly in surgically treated cases, underscores the potential role of local control measures in select patients. This aligns with previous studies indicating that the primary tumor site is an important determinant of prognosis in neuroendocrine neoplasms [13].

However, our correlation analysis revealed that tumor grade, represented by the Ki-67 proliferation index, does not exhibit a significant correlation with either the primary tumor site (r = −0.339, *p* = 0.114) or metastatic burden (r = 0.163, *p* = 0.457). These findings suggest that tumor proliferation, as assessed by Ki-67, may be driven by intrinsic molecular mechanisms rather than by anatomical origin or the extent of metastatic disease. This observation aligns with previous research indicating that high-grade neuroendocrine carcinomas often behave as biologically distinct entities, largely independent of their site of origin. Unlike well-differentiated neuroendocrine tumors, which may retain site-specific characteristics and hormonal functionality, EP-NECs demonstrate a high degree of molecular heterogeneity and aggressive biological behavior, irrespective of their location [43].

Moreover, the absence of a significant correlation between Ki-67 expression and metastatic burden suggests that tumor dissemination in EP-NECs may not be solely driven by proliferative capacity but rather by additional biological mechanisms. It is possible that molecular alterations such as TP53, RB1, and KRAS mutations play a more dominant role in metastatic progression [37]. These findings underscore the necessity of considering broader molecular and genomic factors when assessing tumor aggressiveness in EP-NECs. From a clinical perspective, they highlight the need for a more comprehensive risk stratification approach, integrating genomic and transcriptomic profiling alongside traditional markers like Ki-67 to refine treatment selection, particularly in metastatic cases. Future studies with larger patient cohorts and molecular subtyping may further elucidate the complex interplay between tumor proliferation, metastatic behavior, and clinical outcomes in EP-NECs [13,44].

Several studies have reported that pancreatic NECs tend to have a more aggressive course compared to other anatomical subtypes, often demonstrating lower response rates to chemotherapy and shorter survival durations [15,17]. This aggressive behavior is likely due to underlying molecular differences, as pancreatic NECs frequently harbor mutations in TP53, RB1, and KRAS, distinguishing them from other EP-NEC subtypes [18]. Our findings reinforce these observations, as pancreatic EP-NEC patients in our cohort experienced a median PFS of only 6.2 months, shorter than GU-origin cases.

Survival outcomes in EP-NEC remain suboptimal despite the current treatment strategies. Multiple studies have reported median PFS and OS values in patients receiving platinum-based chemotherapy. To provide a comparative perspective on survival outcomes across different studies, we have compiled relevant data from the literature, including our study, in the table below. This table summarizes the survival rates and treatment responses in metastatic EP-NEC patients, offering insights into prognosis and therapeutic efficacy (Table 5).

The observed benefit of surgical resection in GU-origin tumors further highlights the potential role of multimodal treatment strategies in EP-NEC management. Prior reports suggest that surgical resection can confer a significant survival advantage in select cases, particularly when combined with systemic therapy [43]. However, due to the rarity of EP-NECs, prospective data on the optimal integration of surgery and systemic therapy remain limited. Our findings contribute to this growing body of evidence by demonstrating that surgical resection was associated with a prolonged PFS of 12.4 months in GU-origin tumors, supporting its potential role in appropriately selected patients.

A key prognostic factor identified in our study was the Ki-67 proliferation index, with 75% of patients exhibiting a Ki-67 of >80%, a finding consistent with previous research [13]. High Ki-67 expression was associated with a significantly worse PFS (*p* = 0.032), reinforcing its role in risk stratification and treatment planning. These findings emphasize the need for a more personalized approach to EP-NEC management, particularly for patients with highly proliferative tumors.

Our multivariate analysis further supports the significance of these prognostic factors. The Ki-67 index was identified as the strongest predictor of PFS, with higher values correlating with significantly shorter survival durations. This aligns with prior studies suggesting that highly proliferative tumors exhibit increased resistance to systemic therapies. Surgical resection remained a critical factor, demonstrating an independent association with improved PFS. These findings reinforce the notion that, despite the aggressive nature of EP-NECs, surgery should be considered in carefully selected cases where resection is feasible.

Interestingly, our analysis also indicated a potential role for immunotherapy in delaying disease progression, as patients who did not receive immunotherapy had a significantly worse PFS. While the role of immune checkpoint inhibitors in EP-NECs remains controversial, emerging evidence suggests that specific molecular subgroups may derive benefit from such therapies. Further prospective trials are warranted to evaluate the efficacy of immunotherapy in EP-NEC patients.

Multivariate analysis did not identify any independent predictors of OS. However, numerical trends suggested that cisplatin–etoposide might be associated with better survival compared to carboplatin–etoposide, and surgical intervention showed a potential benefit, though neither reached statistical significance. Interestingly, the Ki-67 index was not an independent prognostic factor, highlighting the complexity of EP-NECs. These findings emphasize the need for larger studies integrating molecular profiling to improve treatment strategies.

The role of radiotherapy in EP-NECs remains controversial. While radiotherapy is a standard treatment for SCLC, its effectiveness in EP-NECs is less well-defined. Some studies suggest that radiotherapy provides a survival benefit in patients who are not candidates for surgery [25]. In a retrospective analysis, localized EP-NEC patients receiving radiotherapy had better local control rates, particularly in esophageal and anal primaries [47]. However, in patients undergoing curative-intent surgery, the addition of radiotherapy did not significantly improve OS. Our study similarly found no statistically significant OS benefit associated with concurrent chemoradiotherapy (CRT) (*p* = 0.581).

Notably, in our cohort, 25% of patients received concurrent CRT, yet this did not translate into a survival advantage compared to chemotherapy alone. These findings are consistent with the existing literature suggesting that the benefit of radiotherapy may be limited to non-surgical candidates [40]. Further studies are warranted to clarify which patient subgroups derive the greatest benefit from radiotherapy.

Despite the encouraging findings regarding multimodal treatment approaches, there remains a critical need for prospective studies to determine the optimal sequencing and combination of therapies in EP-NECs. Our study, like previous analyses, underscores the aggressive nature of these tumors and the necessity of exploring novel therapeutic strategies beyond platinum-based chemotherapy.

Given the aggressive nature and poor prognosis of EP-NECs, there is a growing interest in identifying novel therapeutic targets beyond traditional platinum-based chemotherapy. Emerging evidence suggests that immune checkpoint inhibitors (ICIs) and Delta-like ligand 3 (DLL3)-targeted therapies may offer promising alternatives for a subset of EP-NEC patients.

Recent studies have highlighted the potential of immunotherapy in NENs, particularly in poorly differentiated NECs. While well-differentiated NETs generally exhibit low tumor mutational burden and minimal PD-L1 expression, NECs show increased immune checkpoint activity, making them potential candidates for ICIs [53,54].

ICIs remain a controversial approach for EP-NECs. In our study, three patients who received immunotherapy beyond the third-line setting experienced disease progression as the best response. This observation aligns with findings from previous reports demonstrating the limited benefit of ICIs in EP-NECs, particularly in tumors with low PD-L1 expression [13]. The KEYNOTE-158 trial similarly reported a low response rate (3.7%) with pembrolizumab in neuroendocrine neoplasms [55].

The KEYNOTE-028 trial evaluated pembrolizumab in advanced NENs, reporting limited efficacy, with overall response rates (ORR) of 3.7–10% [56]. Similarly, a phase II trial of spartalizumab (anti-PD-1) showed an ORR of 4.8% in NECs and 7.4% in NETs, with slightly better responses in lung NETs [57]. Combination immunotherapy, such as the SWOG DART trial (ipilimumab + nivolumab), demonstrated higher efficacy in high-grade NECs, with an ORR of 24% [35].

DLL3 is an inhibitory Notch ligand overexpressed in high-grade neuroendocrine carcinomas, including SCLC and some EP-NECs. Rovalpituzumab tesirine, an antibody-drug conjugate targeting DLL3, has shown promising activity in preclinical and early-phase clinical studies for neuroendocrine malignancies. The ORR was 12.4%, with a median overall survival of 5.6 months, slightly improving to 14.3% ORR and 5.7 months OS in DLL3-high patients. Although phase II studies in SCLC have yielded mixed results due to toxicity concerns, DLL3 remains a potential target for future therapeutic development in EP-NECs [36].

These findings suggest that single-agent ICIs have limited efficacy in NENs, but combination strategies or novel immunotherapeutic approaches, such as bispecific antibodies and CAR-T cells, may offer new treatment avenues. Given these results, ICIs may be more effective in specific molecular subgroups rather than as a generalized treatment for all EP-NECs. Future research should focus on biomarker-driven patient selection and combination strategies with DNA repair inhibitors or chemotherapy to enhance the response rates [19].

Molecular profiling has revealed substantial heterogeneity in EP-NECs, suggesting that different biological subtypes may require distinct therapeutic approaches [19]. The classification proposed by Frizziero et al. (2022) [19] categorizes EP-NECs into SCLC-like, non-neuroendocrine cancer-like, and tumor-agnostic groups, each with unique molecular features and potential therapeutic targets. This framework may help refine treatment algorithms:

- SCLC-like EP-NECs, frequently harboring TP53 and RB1 mutations, may benefit from DNA repair-targeted therapies.
- Non-neuroendocrine cancer-like EP-NECs, with frequent KRAS and BRAF mutations, could be targeted with BRAF and MEK inhibitors.
- Tumor-agnostic EP-NECs, characterized by epigenetic alterations, might respond to EZH2 inhibitors [19].

Given the poor prognosis associated with current treatments, alternative strategies such as targeted therapies and novel ICI combinations warrant further investigation. Agents such as AURKA inhibitors (for MYCN-amplified tumors), PARP inhibitors, and epigenetic modulators have shown preclinical promise [19]. However, their clinical efficacy remains to be validated in prospective studies.

Early diagnosis is challenging due to their nonspecific symptoms and rapid progression. Currently, there are no established screening programs for EP-NECs in the general population. However, individuals with certain genetic syndromes, such as Multiple Endocrine Neoplasia type 1 (MEN1), MEN type 2, and Von Hippel-Lindau syndrome, are at increased risk for developing neuroendocrine tumors. For these high-risk groups, proactive surveillance and preventive strategies are recommended. This may include regular imaging studies and biochemical tests to detect tumors at an earlier, more treatable stage. In some cases, prophylactic surgical interventions might be considered to remove at-risk tissues before malignancy develops. Given the aggressive nature of EP-NECs and the lack of specific early detection methods, further research is essential to establish effective screening strategies and preventive measures for both high-risk individuals and the broader population [34,58,59].

This study provides valuable insights into the clinicopathological characteristics, treatment responses, and survival outcomes of EP-NECs. However, certain limitations should be acknowledged. First, the retrospective design and single-center nature of the study may introduce selection bias and limit generalizability. Additionally, the relatively small sample size, inherent to the rarity of EP-NECs, may restrict the statistical power of subgroup analyses. The heterogeneity of tumor origins and treatment modalities further complicates direct comparisons, underscoring the need for larger, multicenter prospective studies. Another limitation is the lack of comprehensive molecular profiling, which could provide deeper insights into tumor biology and treatment responses.

From a clinical perspective, our findings reinforce the importance of a multidisciplinary treatment approach for EP-NECs. While platinum-based chemotherapy remains the standard first-line therapy, its limited long-term efficacy highlights the need for alternative strategies. Surgical intervention, when feasible, appears to confer a survival benefit, particularly in genitourinary-origin tumors. This underscores the need for individualized treatment planning, integrating surgery and systemic therapies to optimize patient outcomes. Additionally, our results indicate that the Ki-67 proliferation index serves as a key prognostic marker, supporting its use in clinical decision-making.

Despite advancements in EP-NEC management, significant knowledge gaps persist. The role of second-line and beyond therapies remains unclear, with limited consensus on the most effective regimens. Immunotherapy has shown limited benefit in this cohort, but biomarker-driven patient selection may help identify responsive subgroups. Furthermore, the biological and molecular heterogeneity of EP-NECs warrants further investigation, as distinct genetic alterations may inform targeted therapy development. The impact of radiotherapy in different EP-NEC subtypes also remains an area for future exploration.

Future research should prioritize large-scale, prospective trials to validate the prognostic factors identified in this study and refine treatment algorithms. The integration of molecular profiling into routine clinical practice could facilitate personalized treatment approaches, potentially improving outcomes. Additionally, emerging therapies, including immune checkpoint inhibitors, DLL3-targeted agents, and novel chemotherapy combinations, warrant further investigation in EP-NEC patients.

Due to its rapid progression and frequent metastatic presentation, a multidisciplinary and biomarker-driven approach is essential for optimizing treatment outcomes. Histopathological confirmation with a Ki-67 index assessment is critical for classification, as patients with a Ki-67 <55% and those with ≥55% demonstrate differing responses to treatment. Molecular profiling should be performed to identify mutations such as a BRAF, ATM, and MSI status, which may guide targeted therapies. FDG-PET/CT imaging is recommended for accurate staging and treatment planning [37,38].

For first-line treatment, platinum-based chemotherapy remains the gold standard. Cisplatin or carboplatin combined with etoposide has shown moderate efficacy, with a median overall survival (OS) ranging between 10 and16 months. Alternative regimens, such as irinotecan-based combinations, have demonstrated comparable outcomes. Immune checkpoint inhibitors (ICIs), while promising in some cancers, have yielded limited benefit in unselected EP-NEC patients, though ongoing trials aim to determine their role in biomarker-selected subgroups [37,60,61].

For second-line treatment, options depend on prior therapy and patient-specific factors. CAPTEM is a preferred regimen in cases with a lower Ki-67 index, offering moderate disease control. Bevacizumab plus FOLFIRI has shown potential benefit in improving the response rates, though more data are needed. Liposomal irinotecan-based therapies are currently under investigation as emerging second-line strategies [16,62].

**Figure 7:**
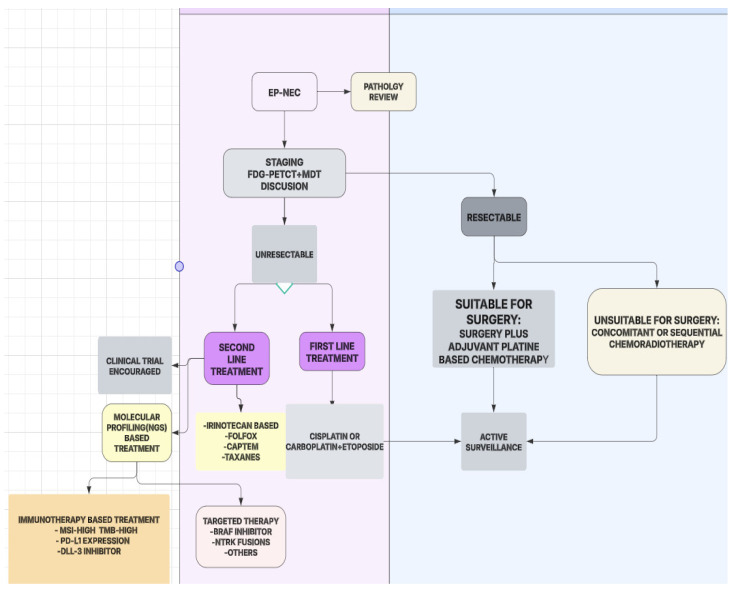
Optimized treatment flowchart for extrapulmonary neuroendocrine carcinoma: MDT: multidisciplinary tumor board, MSI: microsatellite instability, TMB: tumor mutation burden, EP-NEC: extrapulmonary neuroendocrine carcinoma, FDG-PET/CT: fluorodeoxyglucose positron emission tomography/computed tomography.

For localized disease, surgical resection should be considered in selected patients, particularly for gastrointestinal EP-NECs, but the recurrence rates remain high. Adjuvant chemotherapy is recommended in high-risk cases, especially in patients with a Ki-67 >55%, to reduce the recurrence risk [63] (Figure 7).

## 5. Conclusions

EP-NECs remain a rare and highly aggressive malignancy with limited treatment options. Despite achieving a high ORR with platinum-based chemotherapy, the prognosis for EP-NEC patients remains poor, with a median PFS of 8.16 months and a median OS of 14.16 months in our cohort. These findings underscore the urgent need for more effective therapeutic strategies.

Our study highlights the importance of surgical resection in improving survival outcomes. Patients who underwent surgery had significantly longer survival compared to those who did not, suggesting that surgical intervention should be considered in carefully selected cases. However, due to the aggressive nature of EP-NECs, surgical resection is often not feasible, emphasizing the need for additional systemic therapies.

Our results emphasize the importance of considering tumor localization and surgical resection in the treatment decision-making process for EP-NEC patients. Given the significant variation in PFS among different anatomical subtypes, a stratified approach to treatment may be warranted. Future research should explore molecularly targeted therapies and immunotherapy options tailored to distinct EP-NEC subtypes.

Given the poor prognosis associated with current treatment modalities, the development of novel therapeutic strategies is imperative. Multicenter clinical trials, biomarker-driven therapies, and personalized treatment approaches are needed to optimize patient outcomes. Collaborative efforts between oncologists, molecular biologists, and clinical researchers will be essential in advancing the management of EP-NECs and improving survival rates for affected patients.

## Figures and Tables

**Table 1:** Baseline clinic and demographic characteristics of patients.

Variable	(%)
Age	
Median Age (Range)	60.14 (29–82)
ECOG-PS	
0	82.8
1	10.3
2	6.9
Sex	
Female	48.3
Male	51.7
Smoking	
Yes	31.0
No	69.0
Stage at first diagnosis	
Localized	10.3
Locoregional	34.5
Metastatic	55.2
Ki-67	
>80	75.0
60–80	25.0
Surgery	
Yes	31.0
No	69.0
Curative RT	
Yes	39.3
No	58.6
Concurrent CRT	
Yes	25.0
No	75.0
Liver-Directed Therapy	
Yes	6.9
No	93.1
First-line Chemotherapy	
Cisplatin + Etoposide	51.7
Carboplatin + Etoposide	48.3
Second-line Chemotherapy	
Cisplatin + Etoposide	6.9
Irinotecan	17.2
Paclitaxel	3.4
CAPOX	3.4
Third-line Therapy	
Chemotherapy	34.5
Immunotherapy	10.3

CAPOX: oxaliplatin plus capecitabine; RT: radiotherapy; CRT: chemoradiotherapy; ECOG-PS: Eastern Cooperative Oncology Group Performance Status.

**Table 2:** Treatment response rates by therapy line.

Therapy Line	N (%)
1st Line	
Complete Response (CR)	12 (42.9%)
Partial Response (PR)	11 (39.3%)
Progressive Disease (PD)	5 (17.9%)
Objective Response Rate (ORR)	%82.1
Second Line	
Partial Response (PR)	3 (10.3%)
Stable Disease (SD)	1 (3.4%)
Progressive Disease (PD)	7 (24.1%)
Third Line	
Partial Response (PR)	0(0%)
Stable Disease (SD)	2 (6.9%)
Progressive Disease (PD)	3 (10.3%)
Immunotherapy	
Partial Response (PR)	0 (0%)
Stable Disease (SD)	1 (33.3%)
Progressive Disease (PD)	2 (66.6%)

**Table 3:** PFS analysis results.

Variable	PFS Duration (Median, Months)	Univariate *p*-Value	Multivariate HR (95% CI)	Multivariate *p*-Value
Gender		0.747	-	-
Male	8.3 mo.			
Female	8 mo.			
Stage		0.442	-	-
Local	24.2 mo.			
Locoregional	8.1 mo.			
Metastatic	8 mo.			
ECOG-PS		**0.005**	1.452 (0.537–3.926)	0.463
ECOG PS-0	8.1 mo.			
ECOG PS-1	8.3 mo.			
ECOG PS-2	2.1 mo.			
Smoking Status		0.539	-	-
Non-smoker	8.1 mo.			
Smoker	7.1 mo.			
Surgical History		**0.02**	7.291 (1.212–43.862)	**0.03**
No Surgery	8 mo.			
Surgery	NR			
Concurrent CRT		0.847	-	-
No Concurrent CRT	8.3 mo.			
Concurrent CRT	8.1 mo.			
First-line CT (Cis-Eto vs. Carbo-Eto)		0.182	-	-
Cis-Eto:	13.1 mo.			
Carbo-Eto	7.1 mo.			
Ki-67		**0.032**	NE	**0.0**
Ki-67 < 80	36 mo.			
Ki-67 ≥ 80:8 mo.	8 mo.			

CT: chemotherapy; CRT: chemoradiotherapy; Cis-Eto: cisplatin–etoposide; Carbo-Eto: carboplatin–etoposide; ECOG-PS: Eastern Cooperative Oncology Group Performance Status; NR: not reached; NE: not estimable.

**Table 4:** OS analysis results.

Variable	OS Duration (Median, Months)	Univariate *p*-Value	Multivariate Exp(B)	Multivariate *p*-Value
Gender		0.451	-	-
Male	11.8 mo.			
Female	26.4 mo.			
Stage		0.520	-	-
Local	52.2 mo.			
Locoregional	12.4 mo.			
Metastatic	11.8 mo.			
ECOG-PS		0.448	1.106	0.825
ECOG PS-0	13.9 mo.			
ECOG PS-1	NR			
ECOG PS-2	6.5 mo.			
Smoking Status		0.418	-	-
Non-smoker	26.4 mo.			
Smoker	11.7 mo.			
Surgical History		0.385	1.324	0.705
No Surgery	11.8 mo.			
Surgery	26.4 mo.			
Concurrent CRT		0.581	-	-
No Concurrent CRT	14.1 mo.			
Concurrent CRT	14.1 mo.			
First-line CT (Cis-Eto vs. Carbo-Eto)		0.180	0.508	0.331
Cis-Eto:	26.4 mo.			
Carbo-Eto	11.7 mo.			
Ki-67		0.959	1.405	0.645
Ki-67 < 80	26.4 mo.			
Ki-67 ≥ 80:8 mo.	11.8 mo.			

CT: chemotherapy; CRT: chemoradiotherapy; Cis-Eto: cisplatin–etoposide; Carbo-Eto: carboplatin–etoposide; ECOG-PS: Eastern Cooperative Oncology Group Performance Status; NR: not reached.

**Table 5:** Survival data of patients with metastatic gastroenteropancreatic neuroendocrine carcinomas.

Reference	No. of Patients	Cohort	Primary Site	Median PFS (months)	Median OS (months)	2-Year Survival (%)	3-Year Survival (%)
Yao 2008 [5]	2027	All NEC (including Lung)	Mixed	-	5 (4.5–5.5)	-	-
SEER Program 2013 [45]	1389	GEP-NEC	GEP	-	5 (4.7–5.4)	11	8
Sorbye 2013 [46]	252	GEP-NEC (chemotherapy treated)	GEP	-	11 (9.4–12.6)	14	9.5
Sorbye 2013 [46]	53	GEP-NEC (no treatment)	GEP	-	1 (0.3–1.8)	-	-
Machida 2012 [47]	258	GEP-NEC (chemotherapy treated)	GEP	-	11.5	-	-
Bernick 2004 [48]	38	colorectal small-cell NEC	Colon and rectum	-	10.5 (6.7–19)	26	13
Smith 2013 [49]	126	Colorectal NEC	Colon and rectum	-	13	5	-
Fujii 2001 [50]	53	Gallbladder, small-cell NEC (chemotherapy treated)	Gallbladder	-	8	0	-
Strosberg 2011 [51]	32	Pancreatic NEC	Pancreas	-	21	-	-
Garcia-Carbonero 2010 [52]	85	GEP-NEC	GEP	-	1.7	-	-
Celik et al. (2022) [40]	47	EP-NEC (chemotherapy treated)	Stomach (27.6%), Unknown Primary (23.4%), Pancreas (10.6%)	5.83 (4.46–7.20)	13.6 (9.01–18.18)	-	-

NEC, neuroendocrine carcinoma; GEP-NEC, gastroenteropancreatic neuroendocrine carcinoma; SEER, surveillance, epidemiology, and end results.

## Data Availability

The data in this study are available from the corresponding author upon reasonable request.
